# Obtaining Medical Textiles Based on Viscose and Chitosan/Zinc Nanoparticles with Improved Antibacterial Properties by Using a Dielectric Barrier Discharge

**DOI:** 10.3390/polym14194152

**Published:** 2022-10-04

**Authors:** Matea D. Korica, Ana Kramar, Zdenka Peršin Fratnik, Bratislav Obradović, Milorad M. Kuraica, Biljana Dojčinović, Lidija Fras Zemljič, Mirjana Kostić

**Affiliations:** 1 Innovation Center of Faculty of Technology and Metallurgy, University of Belgrade, Karnegijeva 4, 11000 Belgrade, Serbia; 2 Department of Material Science and Engineering and Chemical Engineering, University Carlos III of Madrid, Avda, Universidad 30, 28911 Madrid, Spain; 3 Institute of Engineering Materials and Design, Faculty of Mechanical Engineering, University of Maribor, Smetanova ul. 17, 2000 Maribor, Slovenia; 4 Faculty of Physics, University of Belgrade, Studentski trg 12, 11000 Belgrade, Serbia; 5 Institute of Chemistry, Technology and Metallurgy, University of Belgrade, Njegoševa 12, 11000 Belgrade, Serbia; 6 Faculty of Technology and Metallurgy, University of Belgrade, Karnegijeva 4, 11000 Belgrade, Serbia

**Keywords:** medical textiles, antibacterial properties, viscose, chitosan/zinc nanoparticles, dielectric barrier discharge

## Abstract

This study aimed to obtain functional viscose textiles based on chitosan coatings with improved antibacterial properties and washing durability. For that reason, before functionalization with chitosan/zinc nanoparticles (NCH+Zn), the viscose fabric was modified by nonthermal gas plasma of dielectric barrier discharge (DBD) to introduce into its structure functional groups suitable for attachment of NCH+Zn. NCH+Zn were characterized by measurements of hydrodynamic diameter and zeta potential and AFM. DBD-plasma-modified and NCH+Zn-functionalized fabrics were characterized by zeta potential measurements, ATR-FTIR spectroscopy, the calcium acetate method (determination of content of carboxyl and aldehyde groups), SEM, breaking-strength measurements, elemental analysis, and ICP-OES. Their antibacterial activity was determined under dynamic contact conditions. In addition to SEM, the NCH+Zn distributions on viscose fabrics were also indirectly characterized by measuring their absorbent capacities before and after functionalization with NCH+Zn. Washing durability was monitored through changes in the zeta potential, chitosan and zinc content, and antibacterial activity after 1, 3, and 5 washing cycles. The obtained results showed that DBD plasma modification contributed to the simultaneous improvement of NCH+Zn sorption and antibacterial properties of the viscose fabric functionalized with NCH+Zn, and its washing durability, making it suitable for the production of high-value-added medical textiles.

## 1. Introduction

Rapid medical development and the pursuit of healthcare and the well-being of patients and medical staff have influenced intensive research in many areas. A large amount of research in the area of medical textiles has led to the creation of medical textiles with a wide range of properties [1,2,3,4]. However, the conducted studies were not sufficiently focused on obtaining reusable, i.e., washing-durable, medical textiles [5,6]. This point of view when obtaining medical textiles is very important because the rise in the demand for the medical textile has also increased the amount of medical textile waste, whose remediation is very complex and expensive [7,8,9]. 

Given the prevalence of nosocomial bacterial infections, antibacterial properties are some of the most desirable properties of medical textiles [10,11,12]. Namely, medical textiles represent a potential source of bacterial infections because pathogenic bacteria can be transmitted to them from diseased patients, medical staff, or some other sources, so in contact with contaminated medical textiles, further transmission of pathogenic bacteria can occur [13]. Although viscose is traditionally used for the production of medical textiles, its antibacterial properties do not meet the requirements of modern medical textiles. Due to the fact that viscose, and other textile materials based on cellulose, contain nutrients and energy sources, they are suitable substrates for bacterial growth. Interest in the antibacterial functionalization of viscose especially using natural, non-toxic, biodegradable, and biocompatible antibacterial compounds, such as chitosan and zinc, is growing as the textile industry becomes more oriented towards ecological technologies [2,3,14,15,16,17,18,19]. Recently, different antibacterial chitosan/metal nanoparticles have been prepared, for example, silver/chitosan [20], zinc/chitosan [6], manganese/chitosan [21], gold/chitosan [22], and titanium/chitosan [23]. The advantages of antibacterial applications of chitosan in combination with metals lie in its high surface charge density [21]. Our previous study showed that the chelation of chitosan with zinc increases the positive charge density of chitosan, which leads to the intensification of chitosan’s antibacterial mechanism, which is related to electrostatic interactions, i.e., disrupting the bacterial cell membrane/bacterial cell wall [6]. Studies have shown that the introduction of carboxyl and aldehyde groups in viscose results in improved binding, both in terms of amount and washing durability, of chitosan and zinc to its surface [5,10,11,15]. Carboxyl and aldehyde groups can be introduced into the structure of viscose by various procedures [5,15,24]. However, due to the mentioned current orientation towards ecological technologies and the fact that wet procedures cause serious ecological problems, more and more work is being performed for the introduction of these groups by dry procedures that do not require water, such as the dielectric barrier discharge (DBD) method, which is currently the most promising technique for obtaining plasma at atmospheric pressure [24]. During the DBD treatment, gas molecules become ionized. The resulting gas mixture in the DBD plasma contains reactive species such as energetic electrons, positive and negative ions, neutrals, radicals, and molecular fragments. Generated reactive species are suitable for catalysis of material degradation, building blocks of material assembly, and chemical and physical interactions with molecules on the material’s surface [25,26].

In our previous studies [5,6], two different wet procedures were used to introduce carboxyl and aldehyde groups for improved binding of chitosan (CH)—chitosan nanoparticles with and without incorporated zinc (NCH+Zn and NCH, respectively)—into viscose: TEMPO oxidation and coating with TEMPO oxidized cellulose nanofibrils (TOCN). Since fabrics functionalized with NCH+Zn achieved better antibacterial properties than their counterparts functionalized with CH and NCH, in this study NCH+Zn was chosen for antibacterial functionalization of a viscose fabric. Furthermore, it is important to point out that chitosan and zinc, besides being antibacterial [27,28], also have great antiviral potential [29,30,31,32,33,34]. In particular, recent evidence suggests that chitosan and zinc may play a crucial role in the prevention and treatment of SARS-CoV-2 [35,36,37,38]. This apparent success in the prevention and treatment of SARS-CoV-2 is tempered by the fact that the virus has already mutated into several more infectious, though not equally morbid and fatal, variants. This indicates that SARS-CoV-2 is likely to evolve into an endemic form in the near future [36]. This means that further studies will require continued efforts to prevent and treat SARS-CoV-2 on a seasonal basis and in a manner similar to that we do with seasonal influenza. Such efforts must consider the use of medical textiles with antiviral properties against SARS-CoV-2.

Given the serious ecological problems caused by wet processing and the current orientation of the textile industry towards ecological technologies, in this study carboxyl groups for improved binding of NCH+Zn were introduced into viscose by using DBD before its antibacterial functionalization with NCH+Zn. According to a detailed literature review and the best of our knowledge, modification of a viscose fabric by using DBD, its subsequent antibacterial functionalization with NCH+Zn, and characterization of the obtained product have not previously been reported. Electrokinetic, chemical, morphological, mechanical, and antibacterial properties of unmodified and DBD-modified viscose fabrics before and after antibacterial functionalization with NCH+Zn were studied and compared. 

## 2. Materials and Methods

### 2.1. Materials

Viscose fabric “15A23 viscose uni Sandy–white,” as provided by IGR Agence (Celje, Slovenia), was used as starting material. Chitosan with deacetylation degree 75–85% and low molecular weight (50,000–190,000 Da), 37% hydrochloric acid, sodium tripolyphosphate, zinc acetate dehydrate, calcium acetate, sodium chlorite, 0.1 M sodium hydroxide, sodium hydroxide, potassium chloride, and *n*-heptane were from Sigma-Aldrich (Vienna, Austria), of analytical grade, and used for modification, functionalization, and characterization of viscose fabrics without additional purification. Agar, tryptic soy broth, and yeast extract were from Torlak (Belgrade, Serbia) and used for determination of antibacterial activity of viscose fabrics without additional purification.

### 2.2. Preparation of the NCH+Zn Dispersion

The NCH+Zn dispersion was prepared by a two-phase process. In the first phase, the NCH dispersion was synthesized by ionotropic gelation technique, by dropping the sodium tripolyphosphate solution into the chitosan solution (in a ratio of 1:1 (*w*/*w*)) for 1 h with stirring at room temperature. In the second phase, dissolved zinc acetate was added to the NCH dispersion, resulting in the final synthesis of the NCH+Zn dispersion. The pH of the NCH+Zn dispersion was adjusted to pH 5.5 by the addition of 0.5 M NaOH. Details on the method applied for the preparation of the NCH+Zn dispersion can be found elsewhere [6]. 

### 2.3. Modification of Viscose Fabric by Using a Dielectric Barrier Discharge

In this study, a dielectric barrier discharge with plane-parallel electrodes at atmospheric pressure was used for the modification of viscose fabric. The discharge was generated in a dielectric barrier discharge configuration that consisted of two plan-parallel aluminum electrodes (20.0 × 8.0 cm^2^) covered by a 0.70 mm thick Al_2_O_3_ plate.

The distance between the Al_2_O_3_-covered aluminum electrodes was fixed to 3 mm. To avoid any problem with humidity and to maintain a homogeneous discharge, zeolite in the form of granules was inserted in the discharge area. Details on the dielectric barrier discharge source can be found elsewhere [39]. 

Rectangular fabric samples (19.5 × 7.5 cm^2^, in warp direction) were treated by using a dielectric barrier discharge under the following conditions: 5 kV, 300 Hz, and 600 s. Ambient air was applied as a working gas. 

### 2.4. Functionalization of Viscose Fabrics with NCH+Zn

Unmodified and DBD-modified viscose fabrics were submerged in a NCH+Zn dispersion for 30 min, at room temperature, with a fabric–liquid-bath ratio of 1:50 and 100% wet pick up. Liquid excess was removed onto a laboratory padder (Rapid, Istanbul, Turkey) at a pressure of 2 bar. After that, the fabrics were dried at 40 °C for 30 min in a laboratory oven (Instrumentaria, Zagreb, Croatia).

Before further analyses, the functionalized fabrics were conditioned according to SRPS EN ISO 139:2007 (T = 20 ± 2 °C; RV = 65 ± 4%).

### 2.5. Washing of Viscose Fabrics Functionalized with NCH+Zn

Viscose fabrics functionalized with NCH+Zn were washed according to standard ISO 105-C10 for one, three, or five washing cycles. 

The descriptions and denotations of unmodified and DBD-modified viscose fabrics before and after functionalization with NCH+Zn, and before and after washing, are listed in Table 1.

### 2.6. Characterization of Chitosan/Zinc Nanoparticles

#### 2.6.1. Zeta Potential and Hydrodynamic Diameter Determination

The zeta potential and hydrodynamic diameter of NCH+Zn were determined by electrophoretic and dynamic light scattering, respectively (Zetasizer Nano ZS, Malvern Instruments Ltd., Malvern, UK). The samples were prepared by mixing 2 mL NCH+Zn dispersion and 18 mL distilled water. Data obtained during measurement were processed using Malvern Zetasizer Software, version 7.12.

#### 2.6.2. Atomic Force Microscopy (AFM)

Before AFM analysis, 100 µL of NCH+Zn dispersion in the form of the drop was deposited onto the silicone plate and dried at room temperature. The AFM analysis was performed on an Agilent 5500 AFM multimode scanning probe microscope (Digital Instruments, Santa Barbara, CA, USA) in tapping mode. Silicon cantilevers (ATECNC-20, Nanosensors, Wetzlar, Germany) with a force constant of 12–110 N m^−1^ and a resonance frequency of 210–490 kHz were used for scanning the AFM images.

### 2.7. Characterization of Viscose Fabrics

#### 2.7.1. Zeta Potential Determination

The zeta potential of each of the fabric samples was determined by the streaming potential method, which was performed with a SurPASS electrokinetic analyzer (Anton Paar GmbH, Graz, Austria). Details on the method which was used for the determination of zeta potential can be found elsewhere [5].

#### 2.7.2. Attenuated Total Reflection Fourier Transform Infrared (ATR-FTIR) Spectroscopy

Before ATR-FTIR analysis, the fabric samples were dried at 40 °C for 24 h in a laboratory oven (Instrumentaria, Zagreb, Croatia), and after that stored in a desiccator. ATR-FTIR spectra of fabric samples were recorded by using a Shimadzu IRA Infinity-1 (FT-IR) spectrophotometer (Shimadzu Corporation, Kyoto, Japan) equipped with an attenuated total reflectance accessory (ATR) in the wavenumber range of 600–4000 cm^−1^, at a resolution of 2 cm^−1^, and in 20 scan mode.

#### 2.7.3. Carboxyl and Aldehyde Group Content Determination

Carboxyl and aldehyde group content in the fabric samples was determined by the modified calcium acetate method; details on the method are given in [40]. 

#### 2.7.4. Scanning Electron Microscopy (SEM)

Before SEM analysis, the fabric samples were sputtered with gold using IONSPUTTER, JEOL, model JFC-1100E (JEOL, Tokyo, Japan). SEM analysis was performed with a JEOL JSM-5300 scanning electron microscope (JEOL, Tokyo, Japan). 

#### 2.7.5. Breaking Strength Determination

Breaking strengths of viscose fabrics were determined using a standard fabric constant-rate-of-loading machine (Textest, Schwerzenbach, Switzerland), according to standard SRPS EN ISO13934-1:2008. The fabric samples were cut into rectangular pieces, 50 mm ± 0.5 mm (excluding any fringe). The clamps of standard fabric for the constant-rate-of-loading machine were spaced at 200 mm, and strain rate (bottom clamp rate) was 150 mm/min. Prior to measurements, the fabric samples were conditioned in the relaxed state and tested under the standard conditions (T = 20 ± 2 °C; RV = 65% ± 4%) according to standard SRPS EN ISO 139:2007.

#### 2.7.6. Elemental Analysis

Elemental analysis was performed with a Vario EL III C,H,N,S/O Elemental Analyzer (ElementarAnalysensysteme GmbH, Langenselbold, Germany) to determine the percentages of nitrogen in the fabric samples. Subsequently, the results of the nitrogen percentage were used for the calculation of chitosan content in the fabric samples.

#### 2.7.7. Inductively-Coupled Plasma Optical Emission Spectrometry (ICP-OES)

Before ICP-OES analysis, fabric samples were digested in an advanced microwave digestion system (ETHOS 1, Milestone, Italy). ICP-OES analysis was performed with a Thermo Scientific iCAP 6500 Duo ICP (Thermo Fisher Scientific, Cambridge, UK) spectrometer equipped with RACID86 Charge Injector Device (CID) detector, standard glass concentric nebulizer, quartz torch, and alumina injector. Details on the ICP-OES analysis used to determine the zinc content in the fabric samples can be found elsewhere [6].

#### 2.7.8. The Absorbent Capacity Determination

The absorbent capacity of each fabric sample was determined by the capillary rise method performed on a Krüss Tensiometer K12 apparatus (Krüss GmbH, Hamburg, Germany). The fabric samples were cut into rectangular pieces (2 cm × 5 cm) and hung on the sample holder in the Krüss Tensiometer K12 apparatus. The sample holder was fixed, using the sample cover, into the electronic balance within the tensiometer. The glass vessel, filled with 75 mL of wetting liquid, was placed into the moving table. By starting the experiment, the moving table raised until the tested liquid touched the lower edge of the sample holder. In the moment of the contact, the tested liquid was drawn up through the sample holes, as a result of capillary action. The software monitored the increase in mass of the sample with respect to time during the measurement. The modified *Washburn* equation is used to present the linear dependency of the square height penetration of the penetrating test liquid in the sample *versus* time as:

(1) $$\frac{m^{2}}{t}=\frac{c\rho ɣcos\theta t}{\eta }$$

where the constant *c* is defined as:

(2) $$c=\frac{1}{2}\pi^{2}\tau {\left(\bar{r}\right)}^{5}n^{2}$$

which presents the number (*n*) of microcapillaries and their mean radius ($\bar{r}$) (mm) in bulk material; *m* (g) is the mass of absorbed test liquid; *Ɵ* (°) is the contact angle between tested liquid and solid sample; ρ (g cm^−3^) is the density, ɣ (N m^−1^) is the surface tension, and *η* (Pa s) is the viscosity of the tested liquid.

The constant *c* includes the number of microcapillaries, and it depends on the measured sample and physical properties of the tested liquid. To determine the constant *c*, a measurement was carried out with an optimal wetting (spreading) liquid (in our case *n*-hepane), for which the contact angle *Ɵ* is 0° (cos *Ɵ* = 1). The obtained *c* values for each sample were used in Milli-Q (MQ) water sorption measurements to monitor the weight increase *versus* time (*mass*^2^/*t*) and to calculate the contact angle *Ɵ*. A more detailed explanation can be found elsewhere [41]. The obtained slopes of weight gained due to MQ water penetration as a function of time present the samples’ absorbency rates. The amount of test liquid uptake (g liquid/g sample) at equilibrium express absorbent capacity. The measurements with *n*-heptane and MQ water were performed at least 10 times for each sample in order to obtain statistically significant results.

#### 2.7.9. Antibacterial Activity Determination

The antibacterial activity of fabric samples was determined using a standard method for determining the antimicrobial activity of immobilized antimicrobial agents under dynamic contact conditions ASTM E 2149-01 (2001). According to this standard, the fabric samples show antibacterial activity if the reduction in bacteria is greater than 75%. For antibacterial activity determination, two bacterial strains—Gram-positive bacteria *Staphylococcus aureus* ATCC 25923 (*S. aureus*) and Gram-negative bacteria *Escherichia coli* ATCC 25922 (*E. coli*)—were used.

## 3. Results and Discussion

### 3.1. Characterization of Chitosan/Zinc Nanoparticles

**Figure 1:**
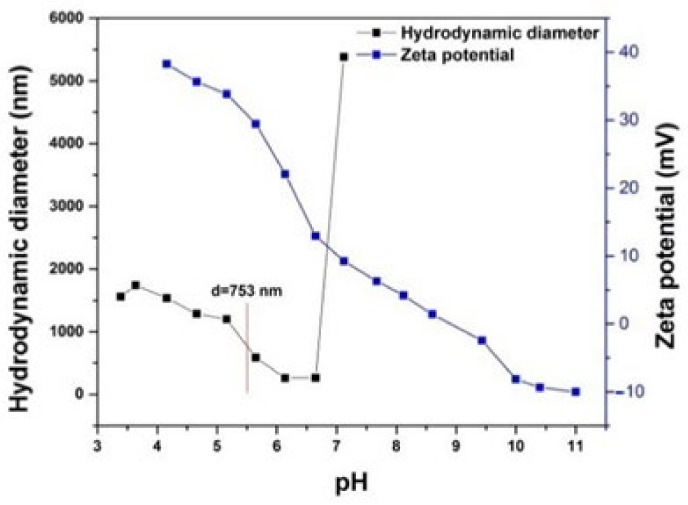
Zeta potential and hydrodynamic diameter of NCH+Zn; modified from [6,11].

The surface charge and hydrodynamic diameter of NCH+Zn are parameters that influence the stability of their dispersion, the interaction of NCH+Zn and viscose fabrics, and the properties of NCH+Zn-functionalized viscose fabrics. The values of zeta potential, as indicators of surface charge and hydrodynamic diameter, are shown in Figure 1.

From Figure 1, it can be seen that the zeta potential of NCH+Zn is higher than ±25 mV at pH values lower than 6. This indicates that the NCH+Zn dispersion is stable at pH values lower than 6 and that its application is possible only under those conditions. 

In the acidic and slightly alkaline pH range, positive zeta potential values were noticed for NCH+Zn due to the protonation of chitosan amino groups and zinc, and suppressed dissociation of chitosan hydroxyl groups, which indicates a positive surface charge of NCH+Zn in this pH range. On the other hand, in the strongly alkaline pH range, negative zeta potential values were noticed for NCH+Zn due to the dissociation of chitosan hydroxyl groups and suppressed protonation of chitosan amino groups and zinc, which indicates a negative surface charge of NCH+Zn in this pH range. In contrast to NCH+Zn, cellulose-based materials exhibit a negative surface charge in the acidic pH range [5]. Bearing in mind that the functionalization of a viscose fabric with NCH+Zn in the acidic pH range can be considered preferable in terms of their mutual interactions. For the functionalization of the viscose fabric with NCH+Zn, pH 5.5 was chosen because it is proven to be the most suitable in terms of simultaneously establishing electrostatic and covalent interactions between a viscose fabric and NCH+Zn [11]. 

The values for the hydrodynamic diameter of NCH+Zn are not the same in the whole pH range, but vary, indicating the aggregation tendency of NCH+Zn in the liquid medium. In the pH range from 3.5 to 6.8, the hydrodynamic diameter of NCH+Zn varies between 1500 and 250 nm, and at pH levels higher than 6.8, the hydrodynamic diameter of NCH+Zn becomes unmeasurable due to the sudden precipitation of NCH+Zn. The sudden precipitation of NCH+Zn in at any pH higher than 6.8 is in accordance with the values of zeta potential (lower than ±25 mV) at pH levels higher than 6.8, which indicates a reduced surface charge of NCH+Zn, i.e., reduced mutual repulsion between NCH+Zn in the liquid medium.

**Figure 2:**
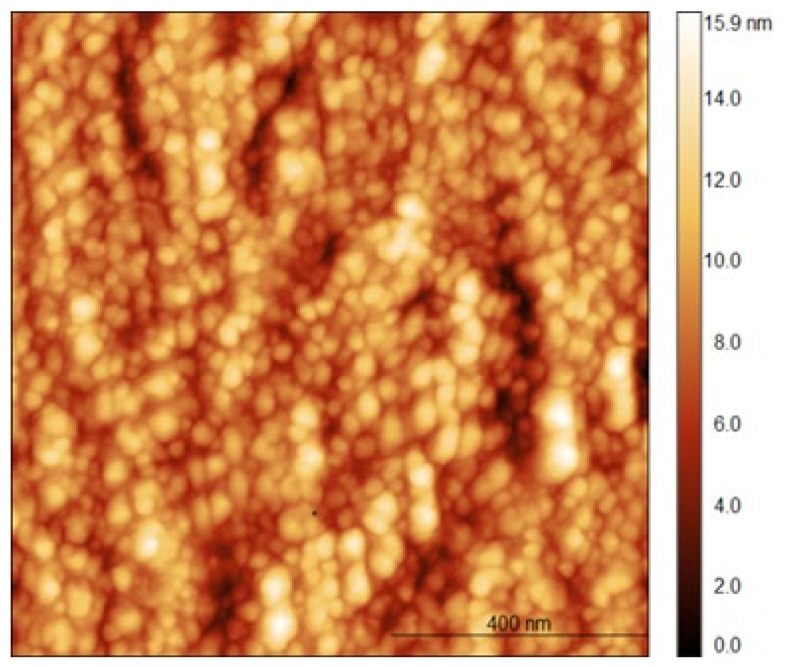
AFM image of NCH+Zn. Image size is 1 × 1 μm^2^. Result previously published in [11].

As it can be seen in Figure 1, the hydrodynamic diameter of NCH+Zn at pH 5.5, at which functionalization of VIS and DBD VIS with NCH+Zn was performed, was 753 nm. Given the pronounced tendency of chitosan to hydration in contact with liquids [42], AFM analysis was applied additionally to determine the dimensions of NCS+Zn in the dry condition. From Figure 2, it can be seen that the dimensions of NCH+Zn in the dry state are about 30 nm. Such a big difference in the dimensions of NCH+Zn in the hydrated state at pH 5.5 (Figure 1) and in the dry state (Figure 2), in addition to their hydration tendency, also confirms their aggregation tendency, which was previously shown by zeta potential and hydrodynamic diameter measurements.

### 3.2. Characterization of Unmodified and DBD-Modified Viscose Fabrics

**Figure 3:**
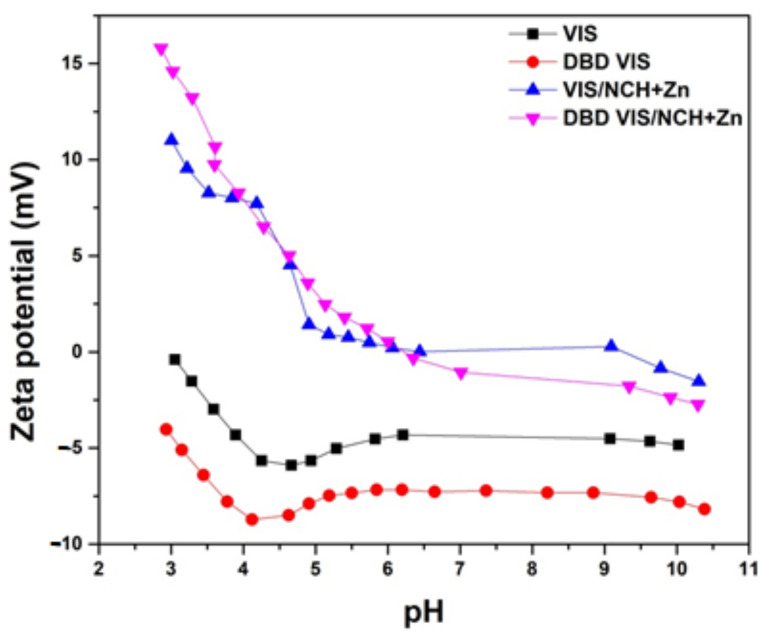
Zeta potential of unmodified and DBD-modified viscose fabrics before and after functionalization with NCH+Zn. Part of results were previously published in [5,6,11].

Modification of viscose by using DBD led to changes of its electrokinetic properties, which can be clearly seen in Figure 3; the pH–zeta-potential curve for DBD VIS was phase-shifted to lower zeta potential values in relation to the pH–zeta-potential curve for VIS, and the isoelectric point for DBD VIS was shifted to a lower pH value in relation to the isoelectric point for VIS. These changes indicate a more negative surface charge of DBD VIS due to the presence of deprotonated acidic groups (COO^−^), making it more suitable for functionalization with NCH+Zn.

**Figure 4:**
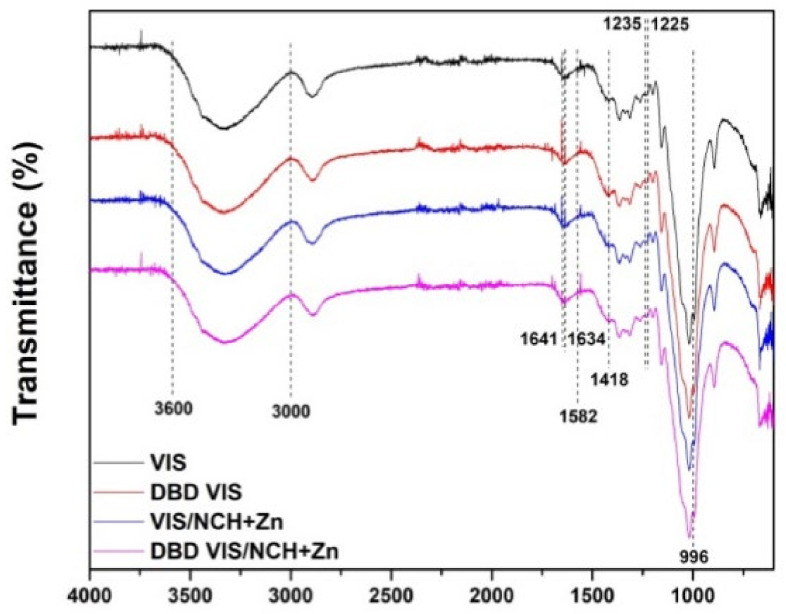
FTIR-ATR spectra of unmodified and DBD-modified viscose fabrics before and after functionalization with NCH+Zn. Part of results previously published in [5,6,11].

In FTIR-ATR spectra of VIS and DBD VIS samples (Figure 4), characteristic bands of cellulose can be observed: related to hydrogen-bonded OH groups at 3000–3600 cm^−1^, C–H stretch in cellulose II and deformation vibrations of CH_2_ and CH_2_OH in cellulose from C6 at 2893 cm^−1^, adsorbed water at 1636 cm^−1^, CH_2_ scissoring at C6 in cellulose II at 1418 cm^−1^, C–H deformation in cellulose II 1372 cm^−1^, CH_2_ wagging vibration at 1316 cm^−1^, O–H in-plain deformation at C6 at 1225–1235 cm^−1^, OH in-plane deformation at 1200 and 1336 cm^−1^, C–O–C asymmetric valence vibration from β-glycosidic linkage in cellulose II at 1158 cm^−1^, C–O vibration mainly from C3–O3H in cellulose II at 1067 cm^−1^, C–O stretching at 1024 cm^−1^, C–O valence vibration at C6 at 996 cm^−1^, and C–O–C valence vibration of β-glycosidic linkage or C1-H deformation in cellulose II at 892–896 cm^−1^ [43,44]. During the DBD modification of VIS, oxidation of its aldehyde and hydroxyl groups into carboxyl groups occurred, wherein the content of aldehyde groups was reduced from 0.018 mmol/g cellulose for VIS to 0.009 mmol/g cellulose for DBD VIS and the content of carboxyl groups increased from 0.064 mmol/g cellulose for VIS to 0.085 mmol/g cellulose for DBD VIS, as determined volumetrically. Changes in the content of aldehyde groups cannot be detected from FTIR-ATR spectra because in the applied conditions the aldehyde groups exist in the partially or totally hydrated forms, and therefore do not show the characteristic band of the aldehyde group [45]. However, slight decreases in the intensity of the bands related to CH_2_ scissoring at C6 in cellulose II at 1418, O–H in-plain deformation at C6 at 1225–1235, and C–O valence vibration at C6 at 996 cm^−1^ indicate the mentioned changes.

**Figure 5:**
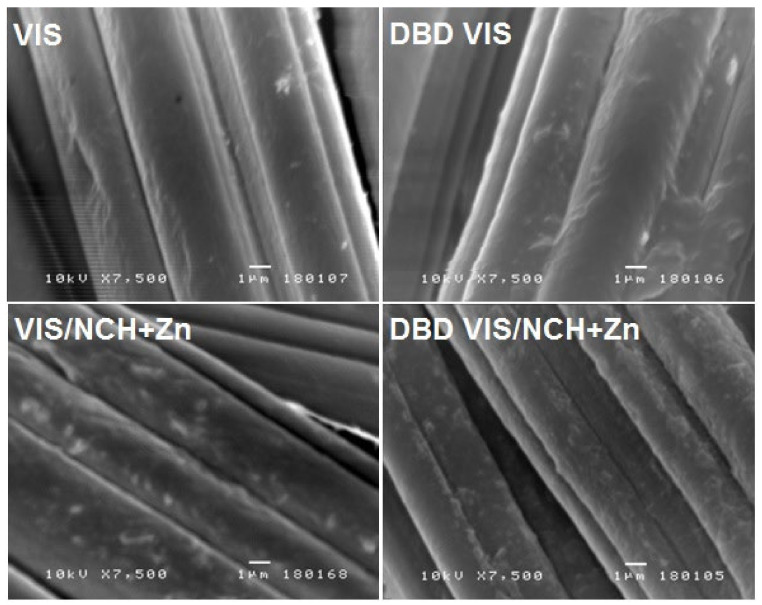
SEM images of unmodified and DBD-modified viscose fabrics before and after functionalization with NCH+Zn.

In addition to changes in electrokinetic and chemical properties, DBD modification also led to changes in the morphology of the VIS fiber surface. It is known that, due to the so-called etching effect, DBD modification leads to cracks and ablation of a viscose fiber’s surface [24], which can be clearly seen in Figure 5. Such morphological changes with a simultaneous increase in the content of carboxyl groups are generally considered very suitable in terms of improving the sorption of cationic molecules and ions [46].

It is known that morphological changes to the fibers after DBD modification usually do not adversely affect the mechanical properties of textiles. Actually, in many cases, there is an improvement [47]. Table 2 shows the effects of DBD modification and NCH+Zn functionalization on the breaking strength of the unmodified and DBD-modified viscose fabrics before and after functionalization with NCH+Zn. Compared to VIS, for DBD VIS, increased breaking strength—by 4.0% in the warp direction and by 3.1% in the weft direction—was noticed. These results can be explained by the increased surface roughness of the DBD-treated viscose (Figure 5) providing more contact points between the DBD-treated viscose fibers and resulting in increased inter-fiber and inter-yarn friction before the occurrence of fabric breakage, and consequently higher breaking strength.

### 3.3. Characterization of Unmodified and DBD-Modified Viscose Fabrics Functionalized with Chitosan Nanoparticles with Incorporated Zinc

Successful functionalization of VIS and DBD VIS fabrics with NCH+Zn was confirmed through remarkable changes in their electrokinetic properties; i.e., a shift of both the whole pH–zeta-potential curves toward higher zeta potential values and their isoelectric points to higher pH values can be observed (Figure 3). Less negative zeta potential values at higher pH indicate smaller numbers of free hydroxyl, carboxyl, and aldehyde groups on the surfaces of VIS/NCH+Zn and DBD VIS/NCH+Zn fabrics due to sorbed NCH+Zn, and more positive zeta potential values at lower pHs indicate suppressed dissociation of free hydroxyl, carboxyl, and aldehyde groups on the surfaces of VIS/NCH+Zn and DBD VIS/NCH+Zn, along with protonation of free amino groups and zinc ions of sorbed NCH+Zn on the surfaces of VIS/NCH+Zn and DBD VIS/NCH+Zn. Lower zeta potential values of VIS/NCH+Zn compared to DBD VIS/NCH+Zn at lower pH values indicate smaller amounts of free amino groups and zinc ions of NCH+Zn, i.e., a smaller amount of sorbed NCH+Zn on the surface of VIS/NCH+Zn.

In the FTIR-ATR spectra of NCH+Zn-functionalized viscose fabrics (Figure 4), slight shifts to higher wavenumbers and lower peak intensity of the broad band related to hydrogen-bonded OH groups at 3000–3600 cm^−1^ can be explained by intermolecular hydrogen bonding between VIS and DBD VIS and NCH+Zn. However, there were no indications of electrostatic interactions of carboxyl groups from VIS and DBD VIS with amino groups and zinc ions from NCH+Zn, nor of covalent interactions of aldehyde groups from VIS and DBD VIS with amino groups from NCH+Zn, which form a Schiff base. The band at 1582 cm^−1^ [48], characteristic for electrostatic interactions, and the band at 1634 cm^−1^ [49], characteristic for covalent interactions by forming a Schiff base, were not noticed and probably were masked by the broad band at 1641 cm^−1^ [50], characteristic for the absorbance of unremovable water bonds to viscose and chitosan [5].

The chitosan and zinc contents in the NCH+Zn-functionalized viscose fabrics were determined by elemental and ICP-OES analyses, respectively. From the presented results (Table 3), it can be seen that the modification by DBD increased the ability of the viscose fabric to bind NCH+Zn (i.e., unmodified viscose fabric bound 640.01 mg chitosan/100 g cellulose and 1858.41 µg zinc/100 g cellulose; DBD-modified viscose fabric bound 1044.60 mg chitosan/100 g cellulose and 2421.50 µg zinc/100 g cellulose). The obtained results are the consequences of chemical and morphological changes in the viscose fiber’s surface after DBD plasma modification. The different ratios of chitosan content to zinc content in VIS/NCH+Zn and DBD VIS/NCH+Zn suggest that the total mass of zinc in VIS/NCH+Zn and DBD VIS/NCH+Zn did not originate only from NCH+Zn. Namely, during the functionalization procedure with NCH+Zn, a certain amount of dissociated zinc ions were directly bound to the VIS/NCH+Zn and DBD VIS/NCH+Zn.

The influences of the surface chemistry and morphology of viscose fabrics modified by DBD plasma on their increased ability to bind NCH+Zn can be also seen in Figure 5. SEM analyses showed that DBD VIS/NCH+Zn fiber surfaces were more densely covered by NCH+Zn than VIS/NCH+Zn fiber surfaces.

The functionalization of VIS and DBD VIS with NCH+Zn also affected their mechanical properties. In Table 2, it can be seen that the breaking strengths (both in warp and weft direction) of VIS and DBD VIS were significantly reduced after their functionalization with NCH+Zn. A decrease in breaking strength by 8.89% in the warp direction and by 10.47% in the weft direction was noticed for VIS after its functionalization with NCH+Zn; a decrease in breaking strength by 9.83% in the warp direction and by 9.64% in the weft direction was noticed for DBD VIS after its functionalization with NCH+Zn. This decrease in the breaking strength after viscose functionalization with NCH+Zn is probably related to chitosan’s interactions with cellulose affecting its fibrillar structure and the hydrolysis of cellulose 1,4-β-glycosidic bonds in the medium acidic conditions used during NCH+Zn functionalization [11].

**Figure 6:**
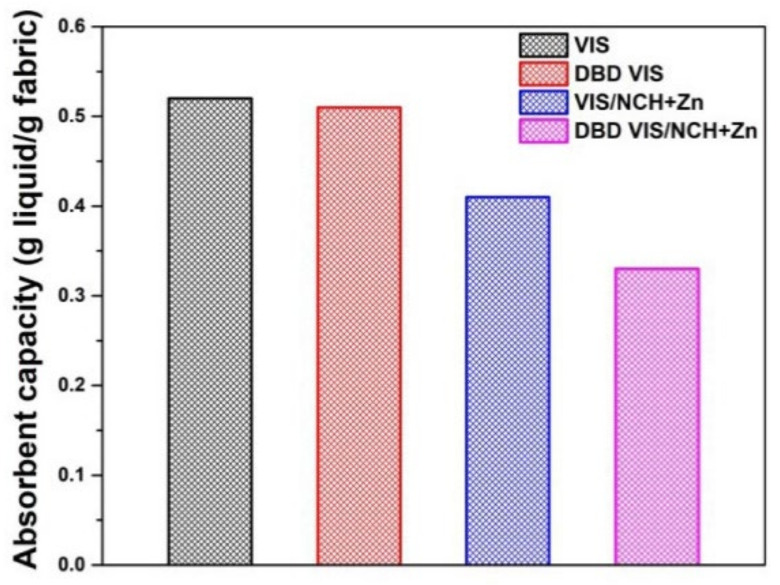
Absorbent capacities of unmodified and DBD-modified viscose fabrics before and after functionalization with NCH+Zn. Part of results previously published in [11].

All mentioned changes should affect the sorption properties of functionalized viscose fabrics. Since chitosan is an extremely hydrophilic molecule [42], it was expected that the absorbent capacities (defined as the amount of liquid uptake by the fabric in an equilibrium state) of VIS and DBD VIS would become higher after their functionalization with NCH+Zn. However, from Figure 6, it can be seen that the absorbent capacities of VIS and DBD VIS became lower after their functionalization with NCH+Zn. It is likely that NCH+Zn, due to their small dimensions (30 nm), occupy the outer pores/cracks of the viscose fibers in VIS/NCH+Zn and DBD VIS/NCH+Zn. In this way, the possibility of penetration of liquid molecules into the amorphous regions of the viscose fibers covered by NCH+Zn is reduced, and the absorbent capacities of VIS/NCH+Zn and DBD VIS/NCH+Zn become lower. It should be pointed out that NCH+Zn were dispersed in the residual chitosan macromolecules’ solution (they were not centrifuged), which probably led to the formation of a chitosan thin-film-coating on the surfaces of VIS/NCH+Zn and DBD VIS/NCH+Zn fibers. This thin-film-coating on the surfaces of NCH+Zn-functionalized fabrics can also reduce the possibility of penetration of liquid molecules into pores/cracks and amorphous regions of the NCH+Zn-functionalized viscose fabrics, and consequently contribute to the additional decrease in the absorbent capacity of VIS/NCH+Zn and DBD VIS/NCH+Zn.

By the same principle, based on the physical barrier, the accessibility of VIS/NCH+Zn and DBD VIS/NCH+Zn fibers nutrients should also be reduced, and thus the growth of bacteria. Further, in our previous study, it was demonstrated that NCH+Zn are a very effective antibacterial agent [6]. Chitosan is known to act against bacteria through four main mechanisms: (1) disruption of the bacterial cell membrane/cell wall, (2) interaction with bacterial DNA, (3) chelation of nutrients required for the existence of bacteria, and (4) formation of an external barrier on the surfaces of bacteria [51]. All of the mentioned mechanisms occur simultaneously, whereby the mechanism based on the disruption of the bacterial cell membrane/cell wall is accepted as the dominant one [52]. Compared to chitosan, NCH+Zn possesses an increased positive surface charge due to the chelation of chitosan with zinc in NCH+Zn. The increased positive surface charge of NCH+Zn ensures the intensification of the antibacterial mechanism based on the disruption of the bacterial cell membrane/cell wall [6]. In other words, due to the increased positive surface charge, NCH+Zn is able to enhance electrostatic interactions with negatively-charged components in the bacterial cell membrane/cell wall. In this way, the blocking of the intra/extracellular exchanges of the bacteria and the leakage of the cytoplasmic content of bacteria are facilitated [11]. 

Zinc is known to act against bacteria through two main mechanisms: (1) disruption of the bacterial cell membrane/cell wall and (2) interaction with bacterial DNA [53]. The synergistic antibacterial activity of NCH+Zn and zinc also occurs due to the release of zinc from NCH+Zn. In addition to the release of zinc from NCH+Zn, the synergistic antibacterial activity of NCH+Zn and zinc is also influenced by the release of zinc directly bound in/on NCH+Zn-functionalized viscose fabrics [6]. From Table 4, it can be seen that the maximum reductions of Gram-positive (*S. aureus*) and Gram-negative (*E. coli*) bacteria by VIS/NCH+Zn and DBD VIS/NCH+Zn were achieved. However, the washing durability of antibacterial activity depends on the releasing of zinc from NCH+Zn and NCH+Zn and zinc from VIS and DBD VIS during washing, which will be discussed in the next section.

### 3.4. Washing Durability of Unmodified and DBD-Modified Viscose Fabrics Functionalized with Chitosan Nanoparticles with Incorporated Zinc

**Figure 7:**
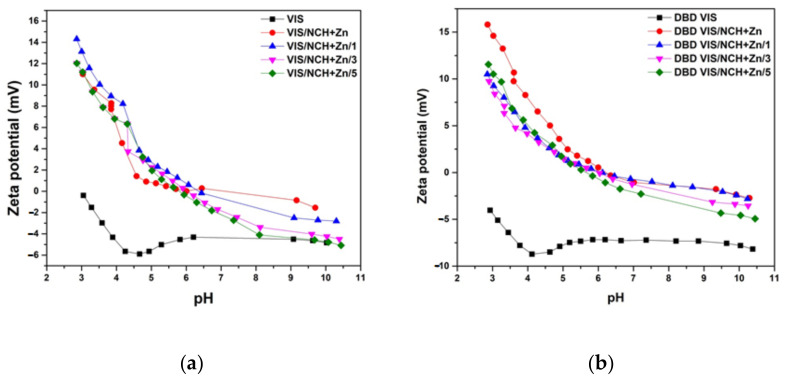
Zeta potentials of unmodified (**a**) and DBD-modified (**b**) viscose fabrics before and after functionalization with NCH+Zn. Part of results previously published in [5,6,11].

Changes in electrokinetic properties of VIS/NCH+Zn and DBD VIS/NCH+Zn fabrics after washing were used to evaluate release of reversibly-bound NCH+Zn and zinc ions during multiple washing cycles (Figure 7a,b). After each washing cycle, their pH–zeta-potential curves were phase-shifted to lower zeta potential values, and isoelectric points were shifted to lower pH values. However, after five washing cycles, pH–zeta-potential curves of VIS/NCH+Zn and DBD VIS/NCH+Zn were not shifted to the pH–zeta-potential curves of VIS and DBD VIS, respectively, indicating that NCH+Zn and zinc ions were still present on VIS/NCH+Zn and DBD VIS/NCH+Zn surfaces.

The release of reversibly-bound NCH+Zn and zinc ions from VIS/NCH+Zn and DBD VIS/NCH+Zn during washing was quantified by determining the chitosan and zinc content after one, three, and five washing cycles (Table 3). For VIS/NCH+Zn, a slight decrease in chitosan content of 9.52% after the first washing cycle, a moderate additional decrease of 19.30% after the third washing cycle, and a significant additional decrease of 30.44% after the fifth washing cycle were determined. In the case of the DBD VIS/NCH+Zn, a moderate decrease in chitosan content of 17.34% after the first washing cycle, a significant additional decrease of 35.48% after the third washing cycle, and a slight additional decrease of 10% after the fifth washing cycle were determined.

Besides a decrease in chitosan content, a simultaneous decrease in zinc content in VIS/NCH+Zn and DBD VIS/NCH+Zn after multiple washing cycles occurred. For VIS/NCH+Zn: the highest decrease of 90.10% was after the first washing cycle, followed by an additional decrease of 8.10% after the third washing cycle and a negligible decrease of 0.71% after the fifth washing cycle. In the same manner, decreased in zinc content occurred for DBD VIS/NCH+Zn: the highest decrease was 94.03% after the first washing cycle, followed by an additional decrease of 4.95% after the third washing cycle, and a negligible decrease of 0.25% after the fifth washing cycle.

If we consider the relative decreases in chitosan and zinc content (52.00% and 99.23%, respectively) in DBD VIS/NCH+Zn after five washing cycles compared with VIS/NCH+Zn (49.21% and 98.90%, respectively), one could conclude that DBD VIS/NCH+Zn has weaker washing durability. However, due to the higher initial chitosan content in plasma-treated samples (1044.60/100 g cellulose and 640.01 mg/100 g cellulose for DBD VIS/NCH+Zn and VIS/NCH+Zn, respectively), the final chitosan content in plasma-treated viscose after five washing cycles was higher (501.40 mg/100 g cellulose and 320.33 mg/100 g cellulose for DBD VIS/NCH+Zn and VIS/NCH+Zn, respectively). This was reflected in antibacterial activity after washing, especially towards *E. coli*, (Table 4), as DBD-treated fabrics retained modest antibacterial activity after three washes, as opposed to untreated ones, which lost activity after the first washing cycle.

The zinc content after washing cycles was relatively similar in all samples, regardless of if they were DBD treated or not, so we can conclude that DBD improves the irreversible binding of chitosan. This influences the improved antibacterial activity towards *E. coli* to a larger extent and towards *S. aureus* to a lower extent. It was shown that DBD treatment also improves antibacterial activity against Gram-positive bacteria after three washing cycles, compared with VIS/NCH+Zn.

It is important to note that the antibacterial activity of both VIS/NCH+Zn and DBD VIS/NCH+Zn fabrics against *S. aureus* was durable for up to five washing cycles. Compared to Gram-positive bacteria, Gram-negative bacteria required a generally higher minimal inhibitory concentration due to the presence of an outer membrane [54,55,56]. That could be the reason why weaker washing durability of antibacterial activity against *E.coli* was achieved for VIS/NCH+Zn and DBD VIS/NCH+Zn. More precisely, for VIS/NCH+Zn, the antibacterial activity against *E. coli* was lost after the first washing cycle, and for DBD VIS/NCH+Zn, the antibacterial activity against *E. coli* was moderate for up to three washing cycles. The more durable antibacterial activity against *E. coli* for DBD VIS/NCH+Zn than for VIS/NCH+Zn can be explained by the higher amount of NCH+Zn on the DBD VIS/NCH+Zn surface after the first washing cycle (Table 3).

Summarized, an obvious correlation between chitosan and zinc content and the antibacterial activity of VIS/NCH+Zn and DBD VIS/NCH+Zn after one, three, and five washing cycles cannot be established. Establishing an obvious correlation is difficult due to the fact that the antibacterial activity of NCH+Zn-functionalized viscose, in addition to the chitosan and zinc content, also depends on the accessibility of NCH+Zn amino groups. NCH+Zn amino groups, which are only accessible in protonated form, can interact electrostatically with the bacterial cell wall, which will lead to antibacterial activity. During washing, with decreases in chitosan and zinc content, changes in the accessibility of the NCH+Zn amino groups are likely to occur. Namely, the bonds between NCH+Zn amino groups, zinc ions, and viscose fabrics are likely reorganized during washing, which results in an unobvious correlation between chitosan and zinc content and the antibacterial activity after washing.

## 4. Conclusions

In this study, modification of a viscose fabric by DBD plasma was applied to introduce functional groups onto its surface, enabling improved binding of NCH+Zn and zinc. The improved binding of NCH+Zn and zinc contributed to improvements in the antibacterial properties of DBD VIS/NCH+Zn. The antibacterial activity against *S.aureus* was durable for up to five washing cycles for bothVIS/NCH+Zn and DBD VIS/NCH+Zn, whereas the antibacterial activity against *E. coli* was lost after the first washing cycle for VIS/NCH+Zn and was moderate for up to three washing cycles for DBD VIS/NCH+Zn. Owing to the achieved antibacterial properties against *S.aureus* and *E. coli*, DBD VIS/NCH+Zn is considered suitable for use in the production of washable medical textiles with extended reusability. In addition, unmodified and DBD-modified viscose fabrics functionalized with NCH+Zn undoubtedly have the potential to contribute to the field of antiviral protection, especially against SARS-CoV-2. Therefore, further investigations of their antiviral activity against SARS-CoV-2 may be of great benefit to future welfare.

## Figures and Tables

**Table 1:** Descriptions and denotation of the fabric samples.

Description of Samples	Washing Cycles			
	0	1	3	5
Unmodified viscose	VIS	-	-	-
DBD-modified viscose	DBD VIS	-	-	-
VIS functionalized with NCH+Zn	VIS/NCH+Zn	VIS/NCH+Zn/1	VIS/NCH+Zn/3	VIS/NCH+Zn/5
DBD VIS functionalized with NCH+Zn	DBD VIS/NCH+Zn	DBD VIS/NCH+Zn/1	DBD VIS/NCH+Zn/3	DBD VIS/NCH+Zn/5

**Table 2:** Breaking strength of unmodified and DBD-modified viscose fabrics before and after functionalization with NCH+Zn. Part of results previously published in [5,11].

Sample	Breaking Strength, N	
	Warp Direction	Weft Direction
VIS	225 ± 1.52	191 ± 3.06
DBD VIS	234 ± 2.83	197 ± 2.83
VIS/NCH+Zn	205 ± 3.11	171 ± 2.36
DBD VIS/NCH+Zn	211 ± 4.91	178 ± 2.27

**Table 3:** The masses of chitosan and zinc in the NCH+Zn-functionalized viscose fabrics. Part of results previously published in [6,11].

Sample	CS, mg/100 g Cellulose	Zn, µg/100 g Cellulose
VIS/NCH+Zn	640.01	1858.41
DBD VIS/NCH+Zn	1044.60	2421.50
VIS/NCH+Zn/1	571.03	184.00
VIS/NCH+Zn/3	459.61	33.54
VIS/NCH+Zn/5	320.33	20.36
DBD VIS/NCH+Zn/1	863.50	144.69
DBD VIS/NCH+Zn/3	557.10	24.84
DBD VIS/NCH+Zn/5	501.40	18.58

**Table 4:** The bacterial reduction by NCH+Zn-functionalized viscose fabrics. Part of results previously published in [6,11].

Sample	Bacterial Reduction, %	
	*S. aureus*	*E. coli*
VIS/NCH+Zn	99.90	99.99
DBD VIS/NCH+Zn	99.99	99.99
VIS/NCH+Zn/1	91.67	4.30
VIS/NCH+Zn/3	86.12	3.85
VIS/NCH+Zn/5	85.83	3.84
DBD VIS/NCH+Zn/1	99.99	98.79
DBD VIS/NCH+Zn/3	99.86	42.31
DBD VIS/NCH+Zn/5	78.51	5.06

## Data Availability

The data presented in this study are available on request from the corresponding author.
